# Single-Cell Transcriptome and Microbiome Profiling Uncover Ileal Immune Impairment in Intrauterine Growth-Retarded Piglets

**DOI:** 10.2174/0113816128411269250707073647

**Published:** 2025-07-17

**Authors:** Yiwen He, Yawei Guo, Xuqing Liang, Hong Hu, Xia Xiong, Xihong Zhou

**Affiliations:** 1 Hunan Provincial Key Laboratory of Animal Intestinal Function and Regulation, College of Life Sciences, Hunan Normal University, Changsha, China;; 2 Institute of Subtropical Agriculture, The Chinese Academy of Sciences, Changsha, China;; 3 Xuanwu Hospital, Capital Medical University, Beijing, 100053, China;; 4 College of Bioscience and Biotechnology, Hunan Agricultural University, Changsha, China;; 5 College of Animal Science and Technology, Hunan Agricultural University, Changsha, China;; 6 Hunan Provincial Key Laboratory of the Traditional Chinese Medicine Agricultural Biogenomics, Changsha Medical University, Changsha, China

**Keywords:** Intrauterine growth retardation, intestine, immune cell, single-cell RNA sequencing, gut microbiota, pseudotime trajectory analysis

## Abstract

**Introduction:**

Impaired intestinal immune function is commonly observed in neonates with intrauterine growth retardation (IUGR), yet its underlying mechanisms and regulatory pathways remain poorly understood. Therefore, we aimed to investigate gene regulatory patterns and microbiota alterations in IUGR piglets.

**Methods:**

Three newborn IUGR piglets and three normal littermates were selected from the same sow and sacrificed at seven days of age. Ileal digesta was collected for 16S rRNA amplicon sequencing (16S-seq), and ileum segments were dissociated for single-cell RNA sequencing (scRNA-seq).

**Results:**

The scRNA-seq results revealed a reduced proportion of plasma B cells in IUGR piglets, along with alterations in the distribution of various T cell subsets. KEGG pathway analysis further indicated a downregulation of the B cell receptor signaling pathway in B cells from IUGR piglets. In contrast, both the T cell receptor signaling pathway and antigen processing and presentation were attenuated in T cells. Pseudotime trajectory analysis suggested that the differentiation of B cells was impaired in IUGR piglets. SCENIC analysis revealed that GATA3, IRF2, and BCL11A were downregulated in T cells of IUGR piglets. The 16S-seq results revealed that α-diversity was lower in IUGR piglets. At the genus level, the relative abundance of *Prevotella* was significantly lower in IUGR piglets.

**Discussion:**

Significant changes were identified in the proportions of B and T cells, their associated signaling pathways, and intestinal microbiota composition in IUGR piglets, suggesting underlying immune dysfunction and dysbiosis.

**Conclusion:**

We identified novel immune-related transcription factors and key microbes as potential therapeutic targets, shedding light on strategies for preventing and treating IUGR.

## 1 INTRODUCTION

Intrauterine growth retardation (IUGR) is associated with impaired general health, particularly deficits in organ development, such as intestinal dysfunction, which leads to increased rates of diarrhea and mortality [1-3]. Approximately 8% of human infants and 20% of newborn piglets are affected by IUGR, presenting a significant challenge to both human and animal health worldwide [4, 5]. The intestine plays a crucial role in digestion, absorption, and immune function. IUGR-related intestinal dysfunction contributes to growth retardation and higher mortality and morbidity rates post-birth [6, 7]. While changes in gut structure, such as reduced mucosal and muscle layer thickness, and in gut function, including decreased enzyme activity related to nutrient digestion and altered hormone secretion profiles, have been observed in IUGR individuals, the precise pathological mechanisms underlying IUGR-induced intestinal diseases, as well as the causal relationship between IUGR and intestinal impairment, remain poorly understood [4].

Additionally, evidence indicates that the intestinal barrier is compromised in IUGR piglets and rats, characterized by increased intestinal permeability, decreased tight junction integrity, and dysregulated microbiota, all of which put the intestinal immune system at risk [8-10]. IUGR-induced intestinal damage triggers an inflammatory response in neonatal piglets at birth [11], and intestinal immune deficiency in IUGR occurs by 7 days of age, as evidenced by a reduced percentage of intraepithelial leukocytes in the duodenum under microscopic examination [12]. However, the alterations in other immune cells and intestinal immune functions remain unclear due to technical limitations.

Single-cell RNA sequencing (scRNA-seq) technology, an advanced biological detection method, has proven highly effective in identifying different cell types, simulating developmental trajectories, and exploring transcriptional patterns [13, 14]. It is increasingly being applied in disease research and animal studies, especially on pigs [15-17]. The cellular changes underlying the pathophysiological condition caused by weaning and the dynamic developmental potential of the neonatal ileum in piglets have been successfully demonstrated using scRNA-seq [18, 19], indicating the feasibility and credibility of scRNA-seq analysis in porcine gastrointestinal research, highlighting the feasibility and reliability of scRNA-seq in this field.

The interaction between the gut microbiota and the intestinal immune system may be affected by IUGR, thereby causing retardation in the development and establishment of immune defense in the intestine [20, 21]. It is reported that the alteration in *Bacteroidetes*, *Bacteroides*, *Escherichia-Shigella*, and *Pasteurella* could be related to nutrient digestion and absorption, and growth and development regulation in IUGR piglets [22, 23]. However, few studies have reported the characteristics of gut microbiome alterations alongside changes in the intestinal immune system. In this study, we performed scRNA-seq analysis on ileal immune cells and 16S rRNA amplicon sequencing (16S-seq) on ileal digesta. We compared KEGG-enriched pathway differences, analyzed pseudotime trajectories of cell differentiation, and explored gene regulatory networks to identify microbiome and immune-related alterations between IUGR and normal piglets.

## 2 MATERIALS AND METHODS

### 2.1 Experimental Design and Sample Collection

A total of 30 sows (Duroc × Landrace × Yorkshire) were monitored for delivery, and one sow was selected as the best candidate based on her litter, which consisted of 11 live-born piglets, including three IUGR piglets (second parity). The criteria for IUGR were based on birth weight < 2 standard deviations below the average birth weight [23, 24]. Additionally, three healthy piglets (with birth weights < 0.5 standard deviations below the average birth weight) were randomly selected for comparison. To eliminate potential external factors, such as inadequate colostrum intake [25], which could influence the experimental results, we ensured that all piglets received proper nursing and had access to fresh drinking water throughout the experiment. At seven days of age, three IUGR piglets (two males and one female; average weight: 1.02 kg) and three healthy piglets (two males and one female; average weight: 1.85 kg) were anesthetized and sacrificed following the procedure as we performed before [26]. Anterior segments of the ileum were collected and temporarily stored in tissue protection buffer (Miltenyi Biotec, cat. 130100008) on ice. Ileal digesta was collected, frozen in liquid nitrogen, and stored at -80°C.

### 2.2 Single-ileal Cell Isolation

Three IUGR samples and three CONT samples were separately pooled and cut into small pieces. A preheated digestion solution (Miltenyi Biotec, cat. 130110204) at 37°C was used at a ratio of 5 mL per cm of ileum segment, and the digestion lasted for 30 minutes. After incubation, 10% FBS (Pricella, cat. 16421050) was added, and the solution was filtered using 40 µm cell strainers. The suspensions were centrifuged at 500 g for 5 minutes at 4°C to remove red blood cells (Roche, cat. 11814389001). The pellets were resuspended in a 0.1% BSA-PBS solution (Sigma, cat. A1933).

### 2.3 Separation of Immune Cells

The immune cells in the prepared single-cell suspension were isolated using a flow cytometer (MoFlo XDP, Beckman) with a CD45 monoclonal antibody (Invitrogen, cat. MA5-28383). The sorted immune cells were counted using Luna-FL (Logos Biosystems, Korea) to meet the standard of ScRNA-seq.

### 2.4 ScRNA-seq and Data Preprocessing

ScRNA-seq was performed using the MobiCube High-throughput Single Cell 3’Transcriptome Set V2.1 (PN-S050200301). The single-cell suspension was adjusted to a concentration of 680-1180 cells/μL and loaded onto a microdroplet formation chip, which was then run on the MobiNova-100 microfluidic platform. Reverse transcription, cDNA amplification, and DNA library construction were performed following the MobiNova protocol. High-throughput sequencing was performed under the PE-150 mode.

The MobiVision software pipeline (version 1.1) provided by MobiDrop was used to demultiplex cellular barcodes. The unique molecular identifier (UMI) count was calculated using the R package Seurat (version 4.0.0) [27]. Low-quality cells and potential multiplets were filtered out based on the following criteria: gene count < 200, UMI count < 1000, and log10GenesPerUMI < 0.7. Additionally, the DoubletFinder package (v2.0.2) [28] was used to eliminate doublets. Normalization of library size was performed using the NormalizeData function in Seurat [27].

### 2.5 Cell Clustering and Sub-clustering

Top variable genes across single cells were selected using the FindVariableGenes function in Seurat [29]. Principal component analysis (PCA) and 2-dimensional Uniform Manifold Approximation and Projection (UMAP) were performed to visualize cell clustering results. The FindAllMarkers function in Seurat was used to identify marker genes for each cell cluster. Cell types were then identified and annotated manually based on marker genes provided in related studies [18, 30-32]. The same procedures were applied for sub-clustering.

### 2.6 Gene Enrichment Analysis

The FindMarkers function in Seurat was used to find differentially expressed genes (DEGs). The thresholds for significant differential expressions were set as *p* < 0.05 and FoldChange > 1.2. KEGG pathway enrichment analysis of the DEGs was performed based on the hypergeometric distribution using R.

### 2.7 Pseudotime Trajectory Analysis

The cell differentiation trajectory was calculated using the Monocle 2 (v2.9.0) package. Genes for cell sequencing were selected using the differentialGeneTest function (ordering gene, *q* < 0.01), the reduceDimension function was used for clustering, and the differentiation trajectory was deduced by the orderCells function [33].

### 2.8 Single-cell Regulatory Network Inference and Clustering (SCENIC) Analysis

SCENIC analysis was performed using the default parameters of the motifs database (RcisTarget) and GRNboost (SCENIC v1.2.4, RcisTarget v1.10.0, and AUCell v1.12.0). Briefly, potential target genes of each transcription factor were first filtered based on co-expression conditions, followed by motif analysis to identify the actual transcription factor and its corresponding target genes. The Regulon Activity Score (RAS) was calculated using the AUCell package (v1.8.0). The Regulon Specificity Score (RSS) and Connection Specificity Index (CSI) of all regulators were calculated with the scFunctions package based on Jensen-Shannon divergence (JSD).

### 2.9 Microbial 16S-seq and Analysis

Ileal digesta DNA was extracted and isolated using the QIAamp DNA Stool Mini Kit (QIAGEN, China). Bacterial 16S rRNA gene sequences (V3-V4 region) were amplified using specific primers 341F: 5’-CCTAYGGGRBGCASCAG-3’ and 806R: 5’-GGACTACNNGGGTATCTAAT-3’. After PCR, the amplicons were sequenced using the Illumina NovaSeq 6000. Analyses such as α-diversity and β-diversity were calculated as we performed before [34, 35]. Briefly, α-diversity (Ace, Chao1, observed species, phylogenetic diversity (PD), Shannon, and Simpson index) was assessed in QIIME 2. The β-diversity was calculated using principal coordinate analysis (PCoA) based on the unweighted UniFrac distance in R. KEGG analysis was conducted using PICRUSt2 software (version 2.3.0). T-tests were performed on SPSS 22.0.

## 3 RESULTS

### 3.1 Clustering and Annotation of Cells Derived From the Ileal Tissue

**Fig. (1):**
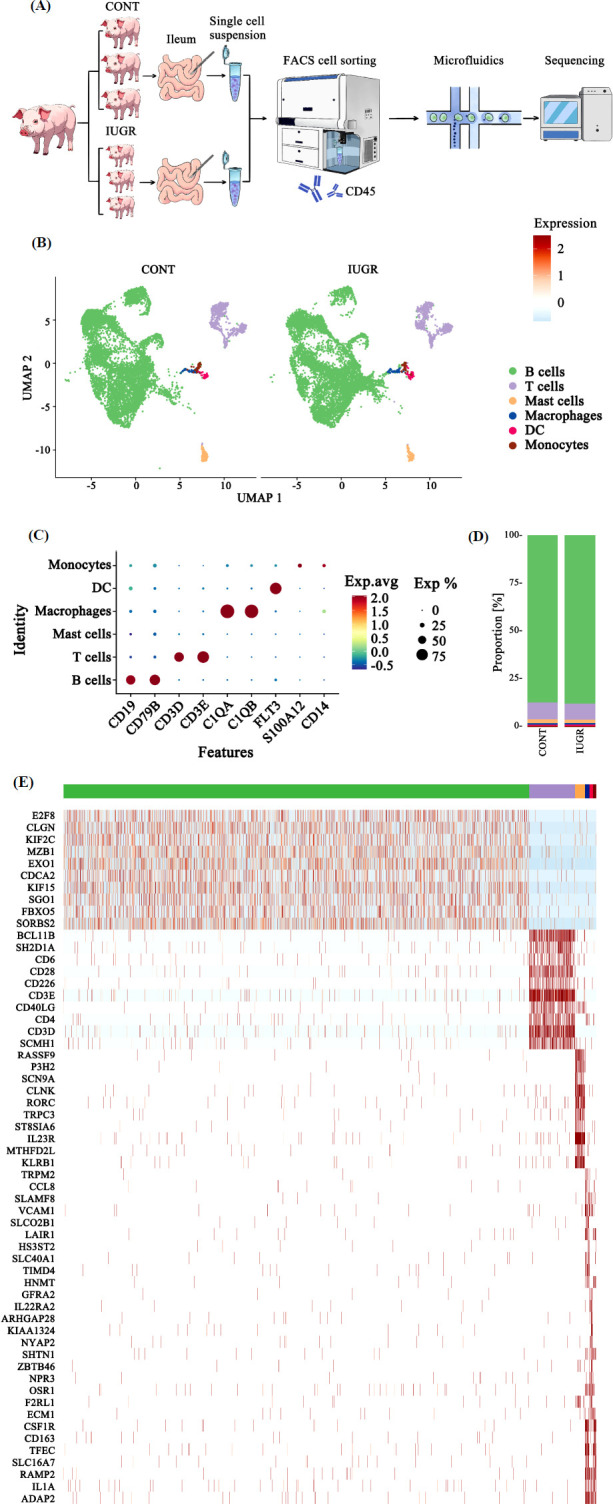
ScRNA-seq profiling of the ileum in CONT and IUGR piglets. (**A**) Flowchart of experiments in this study. (**B**) UMAP visualization of cell-type clusters. (**C**) Dot plot of cluster classic marker genes. (**D**) Proportion of each cell type. (**E**) Top 10 genes for each cluster.

Ileum tissues were dissected for single-cell isolation using a standard protocol. Immune cells were then sorted using flow cytometry, and scRNA-seq was performed subsequently (Fig. **1A**). Following quality control, a total of 17,441 high-quality cells were obtained across all samples. These cells were divided and annotated into six distinct clusters: B cells (CD19, CD79B), monocytes (CD14, S100A12), macrophages (C1QA, C1QB), T cells (CD3D, CD3E), dendritic cells (DCs) (FLT3), and mast cells (Figs. **1B**, **C**). Among these, B cells comprised 87.17% and 87.73%, and T cells accounted for 8.79% and 8.36% in the CONT and IUGR groups, respectively (Fig. **1D**). Other cell types made up less than 2% in both groups. The top 10 marker genes for each cluster further supported the accuracy and reliability of the cluster annotations (Fig. **1E**).

### 3.2 The Heterogeneity and Similarity of B Cell Subtypes

**Fig. (2):**
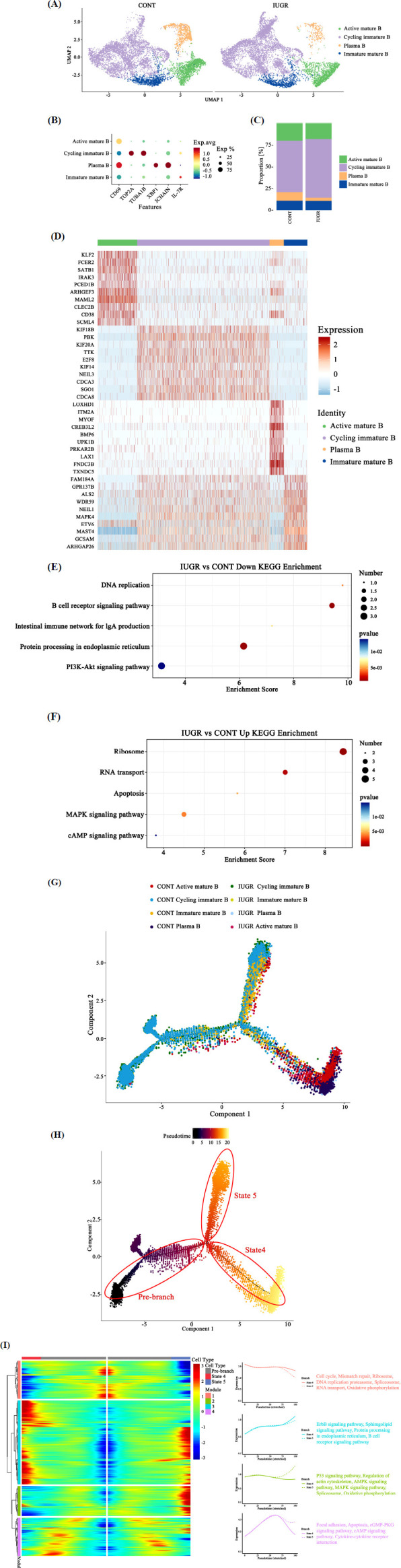
B cell subtypes in the ileum of CONT and IUGR piglets. (**A**) UMAP visualization of B cell subtype clusters. (**B**) Dot plot of subtype cluster classic marker genes. (**C**) Proportion of each cell type. (**D**) Top 10 marker genes for each cluster. (**E**) Downregulated KEGG pathways in B cells. (**F**) Upregulated KEGG pathways in B cells. (**G** and **H**) Differentiation pseudotime trajectory analysis of B cell subtypes. (**I**) The differentially expressed genes along the pseudotime of B cells clustered hierarchically into four modules; the genes of each module are listed in Supplementary File **1**.

Primary analysis of cellular subpopulations and gene expression profiles enabled the classification of potential B cell subtypes. Clustering analysis divided B cells into four subclusters, each characterized by distinct expression signatures: immature mature B cells (IL17R), cycling immature B cells (TOP2A, TUBA1B), active mature B cells (CD69), and plasma B cells (CD69, XBP1, JCHAIN) (Figs. **2A**, **B**). Among these, cycling immature B cells accounted for 59.06% and 67.33%, active mature B cells for 19.75% and 17.93%, immature mature B cells for 11.32% and 11.12%, and plasma B cells for 9.86% and 3.61% in the CONT and IUGR groups, respectively (Fig. **2C**). The top 10 marker genes for each subcluster further validated the reliability and accuracy of the B cell subcluster annotations (Fig. **2D**). KEGG enrichment analysis revealed significant down-regulation of pathways related to DNA replication, B cell receptor signaling, intestinal immune network for IgA production, protein processing in the endoplasmic reticulum, and PI3K-Akt signaling in B cells of IUGR pig ileum. In contrast, pathways such as ribosome, RNA transport, apoptosis, MAPK signaling, and cAMP signaling were significantly upregulated (Figs. **2E**, **F**). To track cell lineage during the differentiation of B cell subclusters, unsupervised pseudotime analysis was performed to identify trajectory bifurcation points. Immature and mature B cells, as well as cycling immature B cells, appeared at the beginning of the trajectory. In contrast, active mature B cells and plasma B cells were located at the terminal state (Figs. **2G**, **H**). Furthermore, we used Monocle2 to identify key molecular changes in a pseudotime-dependent manner, revealing critical processes involved in B cell differentiation. Consistently, a pseudotime trajectory was observed in which immature B cells, mature B cells, and cycling immature B cells differentiated into active mature B cells and plasma B cells (Figs. **2G**, **H**). A total of 2,453 differential genes were identified across four modules with dynamic expression changes along the B cell differentiation trajectory (Fig. **2I**; Supplementary file **1**). Genes in module 1 were associated with the cell cycle and DNA replication, module 2 with P53, AMPK, and MAPK signaling, module 3 with protein processing, ErbB, and B cell receptor signaling pathways, and module 4 with focal adhesion, cytokine-cytokine receptor interaction, and cGMP-PKG signaling, respectively (Fig. **2I**).

### 3.3 Gene Regulatory Networks Identify Key Regulons in B Cells

**Fig. (3):**
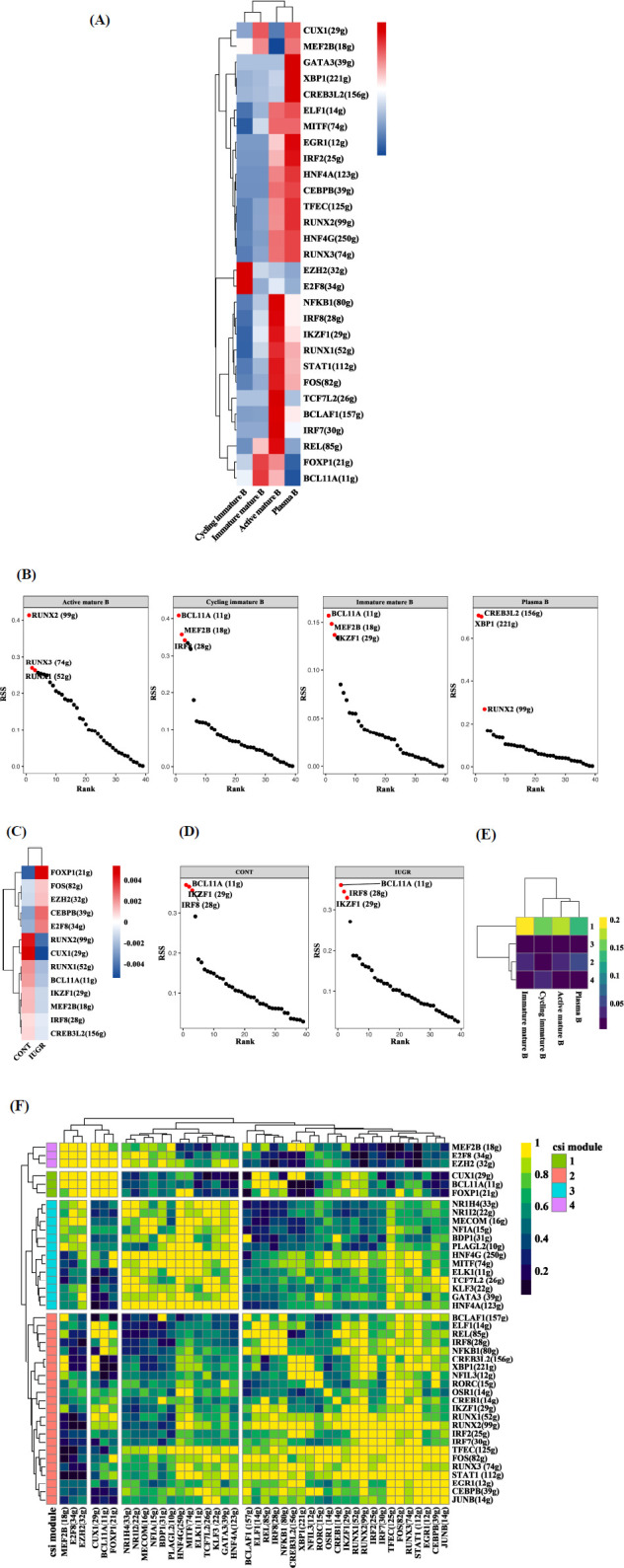
SCENIC analysis of B cell subtypes in the ileum of CONT and IUGR piglets. (**A**) Heatmap of regulon activity score (RAS) in different B cell subtype clusters. (**B**) Ranking plot of regulon specificity score (RSS) in different B cell subtype clusters. (**C**) Heatmap of RAS in different groups. (**D**) Ranking plot of RSS in different groups. (**E** and **F**) Heatmap of regulon connection specificity index (CSI) in different B cell subtype clusters.

Transcription factors (TFs) and their downstream regulated genes form a complex and interrelated network that plays a crucial role in determining and maintaining cell identity. RAS reflects the activity of a regulon, while RSS indicates its specificity. For each B cell subtype, the top three RAS regulons were as follows: EZH2, E2F8, and MEF2B for cycling immature B cells; BCL11A, FOXP1, and CUX1 for immature mature B cells; TCF7L2, IRF7, and BCLAF1 for active mature B cells; and GATA3, CREB3L2, and XBP1 for plasma B cells (Fig. **3A**). The top three RSS regulons for each subtype were RUNX2, RUNX3, and RUNX1 for active mature B cells; BCL11A, MEF2B, and IRF8 for cycling immature B cells; BCL11A, MEF2B, and IKZF1 for immature mature B cells; and CREB3L2, XBP1, and RUNX2 for plasma B cells (Fig. **3B**). When comparing between the two groups, we observed that the RAS of RUNX2, CUX1, RUNX1, BCL11A, IKZF1, MEF2B, IRF8, and CREB3L2 were higher in the IUGR group, while E2F8, CEBPB, EZH2, FOS, and FOXP1 exhibited lower RAS in the CONT group (Fig. **3C**). The top three RSS regulons in both groups were consistent, including BCL11A, IKZF1, and IRF8 (Fig. **3D**). To examine regulon crosstalk, the Regulon-to-Regulon Correlation (CSI) was calculated, ranking regulon significance while mitigating the effects of nonspecific interactions. Hierarchical clustering identified 42 distinct regulons across four major regulon modules. A comparison of regulon activity across these modules revealed that module 1, which includes the TFs CUX1, BCL11A, and FOXP1, exhibited the highest regulatory activity (Figs. **3E**, **F**).

### 3.4 The Heterogeneity and Similarity of T Cell Subtypes

**Fig. (4):**
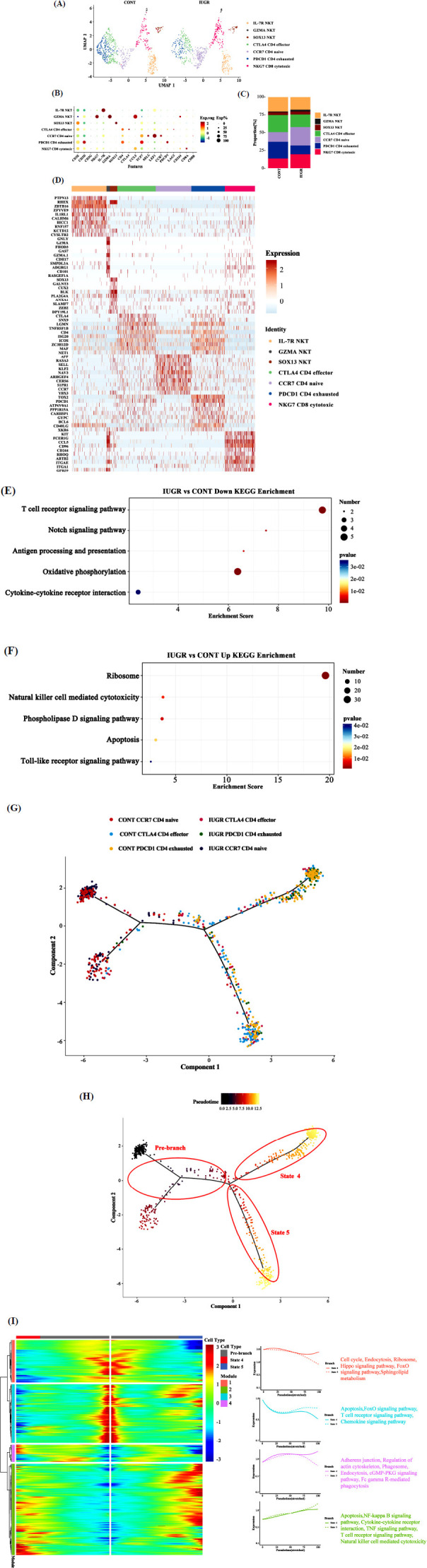
T cell subtypes in the ileum of CONT and IUGR piglets. (**A**) UMAP visualization of T cell subtype clusters. (**B**) Dot plot of subtype cluster marker genes. (**C**) Proportion of each cell type. (**D**) Top 10 marker genes for each cluster. (**E**) Downregulated KEGG pathways in B cells. (**F**) Upregulated KEGG pathways in B cells. (**G** and **H**) Differentiation pseudotime trajectory analysis of T cell subtypes. (**I**) The differentially expressed genes along the pseudotime of T cells clustered hierarchically into four modules; the genes of each module are listed in Supplementary File **2**.

Clustering analysis of T cells identified seven distinct sub-clusters based on their unique expression profiles. These included IL7R NKT cells (IL7R), GZMA NKT cells (NKG7, GZMA), SOX13 NKT cells (CD3E, SOX13), CTLA4 CD4 effector T cells (CD3E, CD4, CTLA4, LAG3), CCR7 CD4 naïve T cells (CD4, CCR7, LEF1, SELL, TCF7), PDCD1 CD4 exhausted T cells (CD3E, CD3D, CD4, TCF7, PDCD1), and NKG7 CD8 cytotoxic T cells (CD3E, CCL5, CD8A) (Figs. **4A**, **B**). The proportions of these sub-clusters were as follows: IL7R NKT cells accounted for 20.20% and 17.75% in the CONT and IUGR groups, respectively; GZMA NKT cells for 1.27% and 2.54%; SOX13 NKT cells for 3.81% and 4.23%; CTLA4 CD4 effector T cells for 24.01% and 17.61%; CCR7 CD4 naïve T cells for 13.60% and 25.92%; PDCD1 CD4 exhausted T cells for 23.51% and 12.39%; and NKG7 CD8 cytotoxic T cells for 13.60% and 19.58% in the CONT and IUGR groups, respectively (Fig. **4C**). The top 10 marker genes for each sub-cluster further validated the reliability and accuracy of the T cell sub-cluster annotation (Fig. **4D**).

KEGG enrichment analysis revealed that several pathways were significantly altered in T cells from the ileum tissue of IUGR pigs. Specifically, the T cell receptor signaling pathway, the Notch signaling pathway, antigen processing and presentation, oxidative phosphorylation, and cytokine-cytokine receptor interaction were significantly downregulated. Conversely, the ribosome, apoptosis, natural killer cell-mediated cytotoxicity, phospholipase D signaling pathway, and toll-like receptor signaling pathway were significantly upregulated (Figs. **4E**, **F**). To track the differentiation of T cell sub-clusters, unsupervised pseudotime analysis was performed to identify bifurcation points in the trajectory. CCR7 CD4 naïve T cells were located at the beginning of the trajectory, while CTLA4 CD4 effector T cells and PDCD1 CD4 exhausted T cells were observed in the terminal states (Figs. **4G**, **H**). Monocle 2 analysis was further employed to identify key molecular changes that occur in a pseudotime-dependent manner and play crucial roles in T cell differentiation. A total of 1,142 differential genes were identified across four modules, with dynamic expression changes along the trajectory of T cells (Fig. **4I**; Supplementary file **2**). Genes in module 1 displayed strong signals related to cell cycle, ribosome, and Hippo signaling pathways; module 2 exhibited prominent signals for NF-kappa B, T cell receptor, and natural killer cell-mediated cytotoxicity signaling pathways; module 3 showed strong signals for apoptosis, FoxO, and T cell receptor signaling pathways; and module 4 highlighted signals for adherens junctions, phagosome, and cGMP-PKG signaling pathways (Fig. **4I**).

### 3.5 Gene Regulatory Networks Identify Key Regulons in T Cells

**Fig. (5):**
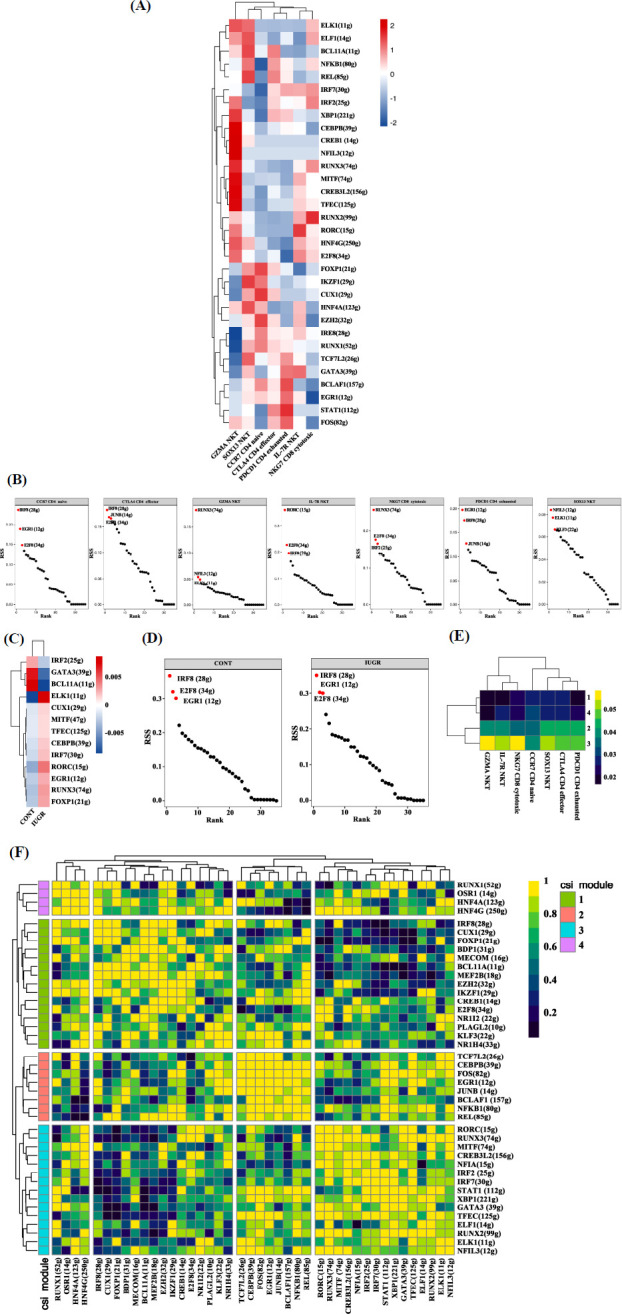
SCENIC analysis of T cell subtypes in the ileum of CONT and IUGR piglets. (**A**) Heat map of regulon activity score (RAS) in different T cell subtype clusters. (**B**) Ranking plot of regulon specificity score (RSS) in different T cell subtype clusters. (**C**) Heat map of RAS in different groups. (**D**) Ranking plot of RSS in different groups. (**E** and **F**) Heat map of regulon connection specificity index (CSI) in different T cell subtype clusters.

The top three regulons with the highest RAS for each subtype are as follows: RORC, GATA, and E2F8 for IL7R NKT cells; NFIL3, CREB1, and CEBPB for GZMA NKT cells; IZKF1, REL, and BCL11A for SOX13 NKT cells; BCL11A, REL, and STAT1 for CTLA4 CD4 effector T cells; CUX1, EZH2, and FOXP1 for CCR7 CD4 naïve T cells; STAT1, BCLAF1, and FOS for PDCD1 CD4 exhausted T cells; and RUNX2, IRF2, and RUNX3 for NKG7 CD8 cytotoxic T cells (Fig. **5A**). The top three regulons with the highest RSS for each subtype are: RORC, E2F8, and IRF8 for IL7R NKT cells; RUNX3, NFIL3, and ELK1 for GZMA NKT cells; NFIL3, ELK1, and KLF3 for SOX13 NKT cells; IRF8, JUNB, and E2F8 for CTLA4 CD4 effector T cells; IRF8, EGR1, and E2F8 for CCR7 CD4 naïve T cells; EGR1, IRF8, and JUNB for PDCD1 CD4 exhausted T cells; and RUNX3, E2F8, and IRF2 for NKG7 CD8 cytotoxic T cells (Fig. **5B**). Between the two groups, RAS levels of IRF2, GATA3, and BCL11A were higher, while ELK1, CUX1, MITF, TFEC, CEBPB, IRF7, RORC, EGR1, RUNX3, and FOXP1 were lower in the CONT group (Fig. **5C**). The top three RSS regulons for both groups were consistent, namely IRF8, E2F8, and EGR1 (Fig. **5D**). Hierarchical clustering of the regulons identified 42 distinct regulons, which were grouped into four major regulon modules. A comparison of the activity levels across these modules revealed that Module 3, which includes RORC, RUNX3, MITF, CREB3L2, NFIA, IRF2, IRF7, STAT1, XBP1, GATA3, TFEC, ELF1, RUNX2, ELK1, and NFIL3, exhibited the highest regulatory activity (Figs. **5E**, **F**).

### 3.6 Alterations in Microbiota Composition

**Fig. (6):**
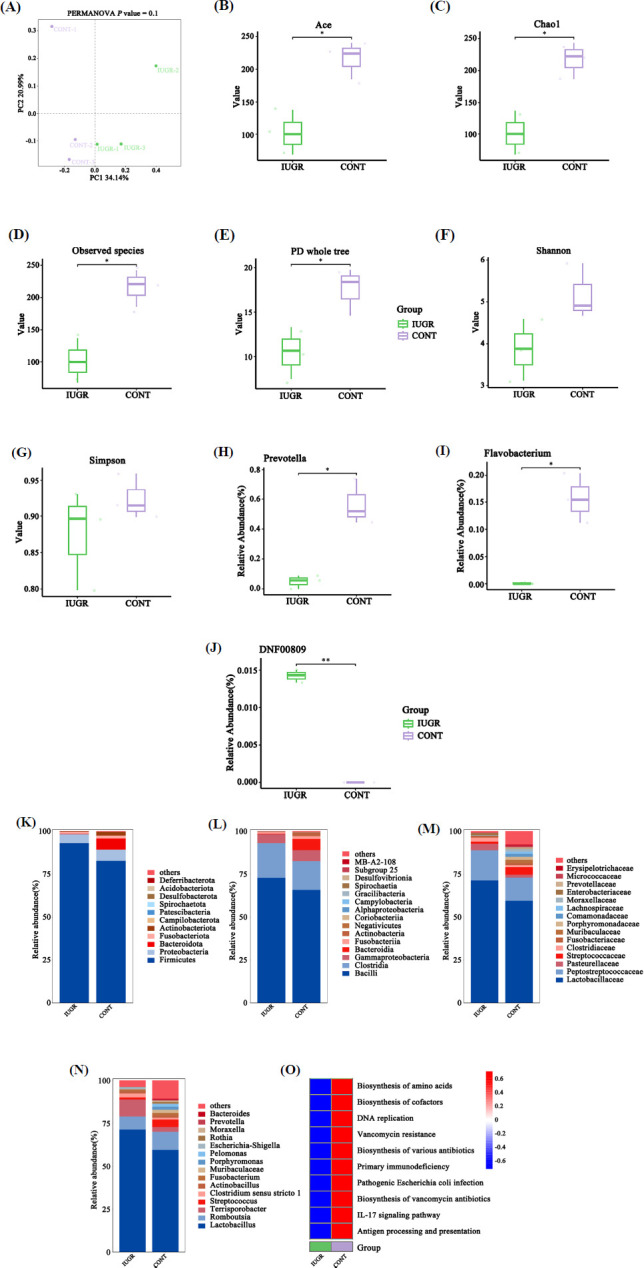
Alterations of microbiota composition in the ileal digesta between CONT and IUGR piglets. (**A**) The principal coordinates analysis. (**B**) The ACE index. (**C**) The Chao1 index (**D**) The observed species index. (**E**) The PD whole tree index. (**F**) The Shannon index. (**G**) The Simpson index. The histogram of the top 15 microbiota at the phylum level (**H**), class level (**I**), family level (**J**), and genus level (**K**). The relative abundance was analyzed using a t-test for *Precotella* (**L**), *Flavobacterium* (**M**), and *bacterium DNF00809* (**N**). (**O**) KEGG pathway prediction of microbial gene function. *p < 0.05 and **p < 0.01.

PCoA analysis showed that β-diversity had no difference between the two groups (Fig. **6A**). However, the ACE index, Chao1 index, observed species index, and PD whole tree index were decreased in the IUGR group (*p* < 0.05), so the Shannon and Simpson indices (*p* > 0.05) (Figs. **6B**-**G**). The dominant bacteria were identified as *Firmicutes, Bacilli, Lactobacillaceae,* and *Lactobacillus* at the phylum, class, family, and genus levels, respectively (Figs. **6H**-**K**). Notably, the relative abundance of *Prevotella* and *Flavobacterium* was significantly lower in IUGR pigs, while *bacterium DNF00809* was significantly higher in IUGR pigs (Figs. **6L**-**N**). KEGG prediction of microbial gene function revealed that immune-related pathways were downregulated in the IUGR groups, including the biosynthesis of various antibiotics, primary immunodeficiency, the IL-17 signaling pathway, and antigen processing and presentation (Fig. **6O**).

## 4 DISCUSSION

Using pig models for human biomedical and physiological studies is gaining consensus, as rodent studies are often confirmed in pigs before being extrapolated to humans [36-38]. This applies particularly to gastrointestinal and metabolic research due to the anatomical and physiological similarities between pigs and humans [4]. The gastrointestinal tract is regarded as the largest immune organ in animals [39, 40]. Using scRNA-seq and 16S-seq, we observed a significant functional shift in the gut microbiome of IUGR piglets and identified six distinct cell types, including B and T cells, which together account for over 95% of the total cell population. Thus, further analyses focused on B and T cells, revealing differences in cell states, functions, and regulatory pathways between the IUGR and healthy piglets. Due to the low proportion of other cell types, further analysis was not conducted for them.

Early B cell development occurs within the intestinal lamina propria [41]. Although a clinical case study reported reduced numbers of B and T cells in cord blood under severe IUGR conditions [42], we observed no difference in the proportions of B and T cells in the ileum between IUGR and normal conditions. This could be attributed to tissue or organ specificity, or potentially to self-recovery, as the immune system in the gut of IUGR piglets may quickly compensate for immunological deficiencies later in life [12]. A previous case report indicated that B-cell differentiation was impaired in a severely IUGR patient [43]. Consistent with this study, our B cell sub-clustering analysis showed that the proportion of plasma B cells was lower in IUGR pigs. Additionally, plasma B cells were located along stage 4 in the trajectory path. At this stage, critical signaling pathways related to B cell differentiation, including cell cycle, DNA replication, B cell receptor signaling, and cytokine-cytokine receptor interactions, were significantly dysregulated under IUGR conditions. Plasma B cells, along with other terminally differentiated B cells, play a crucial role in protecting the host against enteric infections and maintaining intestinal health primarily through the secretion of IgA [44, 45]. The IgA production pathways of B cells play a crucial role in neonatal immunity [46-48]. We observed impaired DNA replication, B cell receptor signaling, and dysfunction of the intestinal immune network for IgA production in the ileum B cells of IUGR piglets. This suggests a disruption in B cell-related immune responses, which may either be a direct consequence of IUGR or a characteristic feature of the condition. The development and function of T cells rely on a well-maintained B cell niche, and disruptions in B cells may lead to similar dysfunctions in T cells [49]. We observed differences in the proportion of T cell subtypes between the IUGR and healthy piglets, along with distinct changes in their trajectory paths, functions, and regulatory pathways. KEGG analysis revealed that T cell receptor signaling, antigen processing and presentation, and cytokine-cytokine receptor interactions were weakened, while apoptosis was increased, suggesting that T cell-related immune functions were also disrupted in the ileum of IUGR piglets.

Gene regulatory networks are governed by the regulation of TFs and their downstream target genes. In a study investigating the immune status of pre-weaning IUGR piglets, increased expression levels of three TFs, including FOXP3, TBET, and GATA3, were observed in the intestines of IUGR piglets [50]. In contrast, we observed that GATA3 expression was downregulated in the T cells of the IUGR piglet ileum, along with the downregulation of IRF2 and BCL11A, and the upregulation of ELK1, RORC, RUNX3, and FOXP1. The discrepancy between our findings and theirs may be due to the different ages of the piglets at the time of analysis. Nevertheless, both studies underscore the pivotal role of GATA3 in regulating T cell development and function in IUGR piglets. Additionally, we found that RUNX2, RUNX1, and CUX1 were downregulated, while FOXP1 was upregulated in B cells of the IUGR piglet ileum. These TFs are critical for regulating various developmental processes, including proliferation, differentiation, apoptosis, and cell lineage specification, suggesting their potential as key factors in preventing or rescuing IUGR [51-53].

The similar β-diversity observed between the two groups may be attributed to their shared living environment and intake of the same colostrum. However, IUGR resulted in significantly lower α-diversity, which may be related to the disorder of the intestinal immune system. This result is consistent with another study, which reported that IUGR piglets exhibited a lower α-diversity of jejunum microbiota at 7 and 21 days of age [22]. KEGG prediction of microbial gene function revealed that the immune function of the gut microbiome in IUGR piglets was weakened. Notably, *Prevotella*, one of the most predominant genera in the intestine of pigs, is associated with preterm birth and low birth weight in humans [54, 55]. In parallel, our results suggest that a decrease in Prevotella may be a characteristic feature of IUGR piglets. *Prevotella* can activate Toll-like receptor 2 and interact with T cells through antigen processing and presentation, stimulating the release of inflammatory factors, such as IL-6, IL-8, and CCL20 [56]. Meanwhile, *Prevotella* has a significant influence on the expression of GATA3 in T cells [57]. Thus, the decreased expression of GATA3 in T cells of IUGR piglets is highly likely related to the decrease of *Prevotella.* However, the underlying mechanisms governing the interaction between intestinal microbiota and host gene expression in IUGR piglets remain to be fully elucidated. A limitation of the present study is the relatively small sample size, primarily due to the difficulty of obtaining a larger number of both IUGR and normal piglets from the same sow. Further research is also needed to clarify how immune responses mediate these host-microbe interactions.

## CONCLUSION

In summary, this study reveals functional impairments in the intestinal immune system of IUGR piglets. Our findings illuminate the gene regulatory signaling networks within the ileum and identify novel transcription factor markers, GATA3 and Prevotella, as potential therapeutic targets, offering valuable insights into the prevention and treatment of IUGR.

## Data Availability

The scRNA-seq raw data have been deposited in the Genome Sequence Archive of the National Genomics Data Center, China National Center for Bioinformation (Beijing), under accession number CRA023399, and are publicly accessible at https://ngdc.cncb.ac.cn/gsa.

## LIST OF ABBREVIATIONS

DEGs: Differentially Expressed Genes
IUGR: Intrauterine Growth Retardation
PCA: Principal Component Analysis
scRNA-seq: Single-cell RNA Sequencing
UMAP: Uniform Manifold Approximation and Projection
